# Commercial Logging and HIV Epidemic, Rural Equatorial Africa

**DOI:** 10.3201/eid1011.040180

**Published:** 2004-11

**Authors:** Christian Laurent, Anke Bourgeois, Mireille Mpoudi, Christelle Butel, Martine Peeters, Eitel Mpoudi-Ngolé, Eric Delaporte

**Affiliations:** * Institut de Recherche pour le Développement (UMR 145), Montpellier, France;; † Projet Presica, Military Hospital, Yaoundé, Cameroon

**Keywords:** HIV, AIDS, epidemiology, prevalence, genetic diversity, risk factors, commercial logging, Africa, dispatch

## Abstract

We found a high seroprevalence of HIV among young women in a commercial logging area in Cameroon. The vulnerability of these young women could be related to commercial logging and the social and economic networks it induces. The environmental changes related to this industry in Equatorial Africa may facilitate HIV dissemination.

More than 20 years after the beginning of the HIV epidemic, the Joint United Nations Programme on HIV/AIDS (UNAIDS) stated that the epidemic was now taking hold in many African countries (*1*). An estimated 25.0–28.2 million persons are already infected in sub-Saharan Africa, accounting for 70% of all infections worldwide, and Africans represent 10% of the world population. AIDS is now the leading cause of death in Africa (2.2–2.4 million deaths in 2003) (*2*). UNAIDS particularly underlined the rapidly rising prevalence in Cameroon, a central African country (4.7% in 1996, 11.8% in 2001) (*1**,**3*). As in many countries, these data come from sentinel surveillance of women attending urban and semi-urban antenatal clinics.

Data from rural areas are scarce, and the dynamics of HIV infection are poorly documented. Travel has been linked to an increased risk among rural populations (*4*). The recent environmental changes related to commercial logging in Equatorial Africa could potentially facilitate HIV dissemination. Commercial logging has led to road construction in remote forested areas, human migration (especially of single men), and develop social and economic networks (including commercial sex work) that support this industry (*5*). In Cameroon, commercial logging has been growing for at least 4 decades. We have previously shown that these environmental changes might represent a risk to human health through exposure to simian immunodeficiency viruses (*6*). We investigated the seroprevalence of HIV, the nature of circulating HIV genetic variants, and factors associated with HIV infection in a logging area of southern Cameroon.

## The Study

A cross-sectional, community-based survey was performed in September 2001 in a remote village where a sawmill and logging camp have been located since 1973 (Nkonzuh, East Province) and also in two neighboring villages (Mboumo and Kompia, 10 km and 30 km from the logging camp, respectively). The three villages are 250 km east of Yaoundé, the capital of Cameroon (Figure). The total population of the three villages has increased since commercial logging began and was estimated at 1,000 inhabitants at the time of the survey (excluding the logging camp). Approximately 200 workers are employed in this industry; approximately half originate from the region. Some workers live in the traditional neighborhoods of Nkonzuh, and a small number live in Mboumo and Kompia; most live in the logging camp. The survey in Nkonzuh was carried out in the traditional neighborhoods but not in the logging camp itself. All inhabitants >15 years of age were asked to participate in the survey during door-to-door visits. After participants gave informed consent, they were interviewed by using a verbal standard questionnaire in French or a local language. The data gathered included the village name, time spent in the village, house number, date of birth or age, sex, ethnic group, marital status, level of education, occupation, and history of blood transfusion, injection, surgery, circumcision or excision, tattoo, and sexually transmitted infections (STI).

**Figure:**
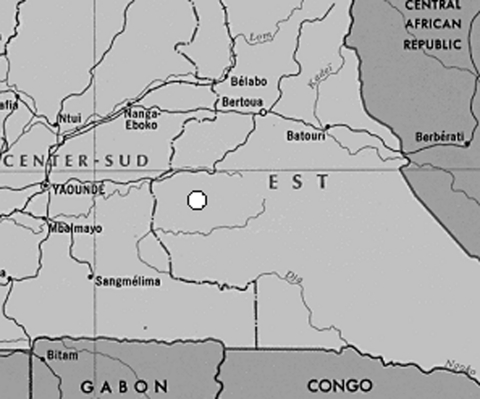
Detail of map of Cameroon, with study area indicated.

Serologic screening for HIV infection was based on an enzyme-linked immunosorbent assay (ELISA) (Murex HIV-1.2.O, Abbott, Rungis, France). All positive samples were confirmed and typed (HIV-1 or -2) by using a line immunoassay (INNO-LIA HIV-1+2, Innogenetics, Ghent, Belgium). All positive samples were further typed (HIV-1 group M, N, O or HIV-2) with an in-house ELISA based on V3 loop peptides. HIV-1–positive samples were genetically characterized in the *gag* and *env* genes by sequencing and phylogenetic analysis, as described (*7*). Syphilis was diagnosed by using the rapid plasma reagin (RPR) (Becton Dickinson, Mountain View, CA) and *Treponema pallidum* hemagglutination (TPHA) (Sanofi Pasteur, Chaska, MN) tests.

The χ^2^ and Fisher exact tests were used to compare the distribution of categorical variables between men and women. For continuous variables, comparisons were based on the nonparametric Mann-Whitney two-sample test. Multivariate random-effects logistic regressions, including sex-specific analyses, were used to identify factors associated with HIV infection (*8*). Independent variables associated with HIV infection, identified by using a conservative threshold of p < 0.25 in univariate analysis, were retained for multivariate analysis. Ninety-five percent confidence intervals (CI) of proportions were estimated by using the binomial exact method.

Four hundred eighty-four persons were enrolled (Table 1). Most (77.8%) were Badjoe, a local ethnic group, and 6.4% were Pygmies; 25 other ethnic groups were also represented. The HIV serologic results were available for 476 persons. Seven persons refused venipuncture after interview, and one sample could not be analyzed. These eight persons did not differ from the other persons in term of sex (50.0% women vs. 47.1% women) but were slightly younger (median, 26.8 years vs. 34.9 years). Five (1.1%) of the 476 HIV serologic results were indeterminate, and these persons were excluded from the analysis of risk factors. The overall HIV seroprevalence was 7.4% (CI 5.2%–10.1%). Women had a far higher HIV seroprevalence than men (overall 11.1% vs. 3.1%) (Table 2), which ranged from 4.9% in women at least 50 years of age to 22.5% in the 25- to 34-year age group. In men, the HIV seroprevalence ranged from 1.4% in the 15- to 24-year age group to 6.0% in the 25- to 34-year age group. The HIV seroprevalence was higher for both sexes, although not significantly, in the village in which the logging camp is located than in the two surrounding villages (Table 1).

**Table 1:** Characteristics of the study population and category-specific HIV seroprevalence in a commercial logging area, southern Cameroon, 2001^a^

Characteristic	Men (N = 228)				Women (N = 256)			
	n	HIV+ (%)	OR	95% CI	n	HIV+ (%)	OR	95% CI
Village of residence								
Nkonzuh	66	6.3	1.00		84	14.5	1.00	
Mboumo	70	4.4	0.68	0.15–3.17	75	6.9	0.44	0.15–1.32
Kompia	92	0.0	–		97	11.3	0.77	0.32–1.86
Median time spent in the village (y)	23				15.2			
Ethnic group								
Badjoe	184	2.7	1.00		190	11.2	1.00	
Pygmies	18	0.0	–		13	0.0	–	
Others	25	8.3	3.18	0.58–17.39	51	14.0	1.28	0.51–3.21
Age group (y)								
15–24	73	1.4	0.89	0.05–14.46	71	10.0	2.11	0.59–7.54
25–34	51	6.0	3.96	0.40–39.26	51	22.5	5.65	1.68–18.94
35–49	39	5.1	3.54	0.31–40.48	52	11.5	2.48	0.66–9.25
>50	65	1.6	1.00		82	4.9	1.00	
Marital status								
Married	124	3.3	1.00		139	4.4	1.00	
Free union	16	6.3	1.95	0.20–18.62	22	36.4	12.19	3.70–40.22
Single	64	1.6	0.48	0.05–4.38	38	18.4	4.82	1.51–15.35
Divorced	8	12.5	4.88	0.47–50.60	8	25.0	7.11	1.18–42.91
Separated	4	0.0	–		11	18.2	4.74	0.83–26.93
Widowed	10	0.0	–		37	8.1	1.94	0.46–8.17
Level of education								
Never schooled	22	0.0	–		68	2.9	1.00	
Primary school	152	2.0	1.00		156	12.4	4.64	1.05–20.54
Secondary school or higher	54	7.7	4.17	0.90–19.31	32	22.6	9.48	1.84–48.85
Occupation								
None	2	0.0	–		21	5.0	1.00	
Culture	126	2.4	1.00		206	11.8	2.56	0.33–20.01
Hunting	29	3.7	1.56	0.03–20.31	0	–	–	
Retired	6	0.0	–		1	0.0	–	
Other	57	5.5	2.44	0.31–18.74	25	12.5	2.71	0.26–28.37
Potential risk factors for HIV infection								
Blood transfusion	2	0.0	–		10	33.3	4.32	1.02-18.35
Injection	220	3.2	–		248	11.0	0.75	0.09–6.47
Surgery	31	3.2	1.02	0.02–8.89	39	5.3	0.40	0.09–1.75
Circumcision	220	3.2	–		–	–		
Excision	–				0	–		
Tattoo	10	10.0	3.80	0.41–34.94	28	17.9	1.88	0.65–5.42
Sexually transmitted infection	90	5.6	3.87	0.73–20.40	37	21.6	2.66	1.07–6.60
Serologic evidence of syphilis^b^	19	5.3	1.93	0.22–16.99	27	7.4	0.61	0.14–2.71

^a^+, positive; OR, odds ratio; CI, confidence interval. ^b^Rapid plasma reagin and *Treponema pallidum* hemagglutination positive.

**Table 2:** Seroprevalence of HIV infection according to sex and age in a commercial logging area, southern Cameroon, 2001^a^

Age groups (y)	Men			Women			Both	
	No. tested	HIV+ (%)	95% CI	No. tested	HIV+ (%)	95% CI	OR^b^	95% CI
15–24	71	1.4	0.1–7.6	70	10.0	4.1–19.5	7.78	0.93–64.98
25–34	50	6.0	1.3–16.6	49	22.5	11.8–36.6	11.38	0.79–163.10
35–49	39	5.1	0.6–17.3	52	11.5	4.4–23.4	3.25	0.30–35.41
>50	64	1.6	0.1–8.4	81	4.9	1.4–12.2	3.26	0.36–29.95
Total	224	3.1	1.3–6.3	252	11.1	7.5–15.7	4.39	1.74–11.08

^a^CI, confidence interval; OR, odds ratio. ^b^HIV prevalence in women versus men.

All 35 seropositive persons were infected by HIV-1, and no one was coinfected by HIV-2. Samples from 28 persons reacted with group M peptides, and two others reacted with both group M and O peptides. Five serum samples did not react with group M, N, or O peptides. Twenty-six of the 35 seropositive samples could be amplified, and all were genetically characterized, in both *gag* and *env* (n = 24), *gag* only (n = 1), or *env* only (n = 1). The circulating recombinant form (CRF) 02_AG strain predominated (72.0% in *gag* and 76.0% in *env*), and several other variants cocirculated (subtypes A, F2, G, and H and CRF06_cpx and CRF11_cpx). A discordant profile was observed between the *gag* and *env* genes in three persons (12.5%): A*^gag^*/H*^env^*, G*^gag^*/CRF06_cpx*^env^*, G*^gag^*/CRF11_cpx*^env^*, respectively.

In univariate analysis, HIV infection in women was associated with age group (p = 0.03), marital status (p = 0.002), level of education (p = 0.03), history of blood transfusion (p = 0.05), and STI (p = 0.04) (Table 1). In men, no factors were associated with HIV infection. In multivariate analysis, HIV infection remained strongly associated with sex (odds ratio 10.22; CI 3.19–32.80; p < 0.001), after adjustment for marital status, level of education, and history of STI. No specific risk factors were found in men. In contrast, women who are unmarried, educated, or have a history of an STI were more likely to be infected by HIV than women who were married, never-schooled, or did not have a history of an STI (Table 3).

**Table 3:** Multivariate analysis of factors associated with HIV infection among women living in a commercial logging area, southern Cameroon, 2001^a^

Variable	Adjusted OR^b^	95% CI^b^
Marital status		
Married	1.00	
Free union	10.85	3.10–37.92
Single	4.33	1.31–14.33
Divorced	10.78	1.55–75.09
Separated	3.73	0.59–23.41
Widowed	5.91	1.01–34.44
Level of education		
Never schooled	1.00	
Primary school	6.04	0.97–37.49
Secondary school	10.17	1.27–81.45
History of STI		
No	1.00	
Yes	3.14	1.12–8.81

^a^OR, odds ratio; CI, confidence interval. ^b^Initial model included village of residence, time spent in the village, age group, marital status, level of education, and history of blood transfusion, surgery, tattoo and sexually transmitted infection (STI). ORs compare each category individually to the first category.

## Conclusions

We identified a population with a high seroprevalence of HIV infection; nearly one quarter of women 25–34 years of age were infected. The HIV seroprevalence among women 15–44 years of age (median 26 years) was slightly higher than among women of the same age group (median 22 years) who attended urban and semiurban antenatal clinics in the East Province (14.5% vs. 10.0%) (9). HIV seroprevalence among women was comparable in the 15- to 24-year age groups (10.0% vs. 10.4%) and the 35- to 44-year age groups (11.5% vs. 12.5%), while it was much higher in the 25- to 34-year age group (22.5% vs. 8.3%). Lower seroprevalence rates among women who went to the antenatal clinics than in the general female population have been reported in several African countries, which is attributable to lower fertility among HIV-infected women (*10*), but the far higher rate observed in our 25- to 34-year age group is particularly striking.

The overall HIV seroprevalence was higher, although not significantly, in our survey (7.4%, CI 5.2%–10.1%) than in another survey conducted in villages of the same province (4.5%, CI 3.3%–6.1%) (*11*). The villages we surveyed are more readily accessible by car, which favors travels to and from places with higher HIV seroprevalence (towns and other regions). The proportion of Pygmies, who are known to have a low HIV seroprevalence (*12*), confirmed by our results, is lower in the area we surveyed. Some villages surveyed by Nyambi et al. (*11*) were located in an area with a more recent history of logging activity where environmental changes had not yet fully affected the epidemic.

The high HIV seroprevalence in women 25–34 years of age living in this rural area could be related to commercial logging. In a context in which workers had relatively high salaries (U.S. $60 to U.S. $530 per month), sexual networks were extensive and complex (*13*). An estimated 40 female sex workers were permanently living in the logging camp (S. Loul, pers. comm.). In addition, ≈100 women arrived at the logging camp from towns or neighboring villages at the time of salary distribution (twice a month), to trade or offer paid sex (U.S. $1.50 per intercourse). Some men and women had sex with several partners a night. Some workers’ wives also had extramarital sex. Seroprevalence in both sex and odds ratio when men and women are compared are age-specific; seroprevalence in women 25–34 years of age is greater for those in our study than those in the sentinel surveillance. The lack of association with local risk factors, such as blood transfusion and injection, and the results of the multivariate analysis suggest that young, unmarried, and educated local women could be mainly infected by workers during unprotected relationships in exchange for money or goods. The high prevalence of syphilis confirmed high-risk sexual behavior (11.8% in women 15–44 years of age compared to 3.6% among those who attended antenatal clinics) (*9*).

HIV-1 genetic diversity and its distribution were similar to that observed in towns (*7**,**14*), which suggests that the spread of HIV in this rural area results from numerous introductions of the virus. The vulnerability of this rural population, especially young women, to HIV infection could be related to commercial logging and the social and economic networks it creates.
